# Risk estimation before progression to mild cognitive impairment and Alzheimer’s disease: an AD resemblance atrophy index

**DOI:** 10.18632/aging.102184

**Published:** 2019-08-29

**Authors:** Lei Zhao, Yishan Luo, Darson Lew, Wenyan Liu, Lisa Au, Vincent Mok, Lin Shi, for the Alzheimer Disease Neuroimaging Initiative

**Affiliations:** 1 BrainNow Research Institute, Shenzhen, Guangdong Province, China; 2 Department of Medicine and Therapeutics, The Chinese University of Hong Kong, China; 3 Therese Pei Fong Chow Research Centre for Prevention of Dementia, The Chinese University of Hong Kong, China; 4 Chow Yuk Ho Technology Centre for Innovative Medicine, The Chinese University of Hong Kong, China; 5 Lui Che Woo Institute of Innovative Medicine, The Chinese University of Hong Kong, Shatin, China; 6 Department of Imaging and Interventional Radiology, The Chinese University of Hong Kong, Shatin, China; 7 Data used in preparation of this article were obtained from the Alzheimer’s Disease Neuroimaging Initiative (ADNI) database (adni.loni.usc.edu). As such, the investigators within the ADNI contributed to the design and implementation of ADNI and/or provided data but did not participate in analysis or writing of this report. A complete listing of ADNI investigators can be found at: http://adni.loni.usc.edu/wp-content/uploads/how_to_apply/ADNI_Acknowledgement_List.pdf

**Keywords:** atrophy index, Alzheimer’s disease, biomarker, conversion, automated brain volumetry

## Abstract

To realize an individual-level risk evaluation of progression of early Alzheimer’s disease (AD), we applied an AD resemblance atrophy index (AD-RAI) to differentiate the subjects at risk of progression from normal subjects (NC) to mild cognitive impairment (MCI) and from MCI to AD. We included 183 subjects with a two-year follow-up: 50 NC stable (NCs), 23 NC-to-MCI converters (NCc), 50 MCI stable (MCIs), 35 MCI-to-AD converters (MCIc), 25 AD stable (ADs). ANCOVA analyses were used to identify baseline brain atrophy in converters compared with non-converters. To explore the relative merits of AD-RAI over individual regional volumetric measures in prediction of disease progression, we searched for the optimal cutoff for each measure in logistic regressions and plotted the longitudinal trajectories of these brain volumetric measures in converters and non-converters. Baseline AD-RAI performed the best in differentiating NCc from NCs (odds ratio 26.35, AUC 0.740) and MCIc from MCIs (odds ratio 8.91, AUC 0.771). The AD-RAI presented greater increase in the second year for NCc vs. NCs but not for MCIc vs. MCIs. Baseline AD-RAIs were also associated with CSF-based and PET-based AD biomarkers. These results showed the potential of AD-RAI in early risk estimation before progression to MCI/AD at an individual-level.

## INTRODUCTION

As the leading cause of dementia, Alzheimer's disease (AD) poses significant challenges in the cost of medical care and associated societal burdens. The prevalence of AD is still increasing dramatically with ageing population worldwide, because the primary risk factor of AD is old age [1]. As several recent Phase 3 trials of mild-to-moderate AD have failed [2–4] and no effective disease-modifying treatments for AD patients are currently available, it is critical to identify biomarkers that specify early stages of AD and facilitate early interventions [5, 6] before significant neuronal damage. Among the biomarkers of prodromal AD, neuroimaging measures have been playing a central role in monitoring disease progression [7]. One of the more common types of neuroimaging data is structural magnetic resonance imaging (MRI) that identifies brain atrophy [8], which has been widely studied to predict disease progression for AD.

To monitor the disease progression of AD with brain atrophy measures, most researchers focused on identifying the mild cognitive impairment (MCI) subjects at risk of progression to AD, and many achieved good classification performance in terms of individual diagnosis using machine learning models [8–14]. Some studies also investigated the probability of even earlier prediction of AD conversion, and they found that brain atrophy (e.g. in hippocampal volume) could even identify healthy subjects up to 10 years before their onset of AD [15, 16]. However, the findings of these studies were based on group comparisons between converters and non-converters, which could not be applied to classify specific healthy individuals at risk of AD. In fact, the sample sizes of the studies targeting at healthy subjects till AD conversion are generally small, due to the long period of follow-up to capture a sufficient number of converters. In this regard, it might be favorable to monitor disease progression of AD for individuals in two separate periods: identifying healthy subjects at risk of MCI and identifying MCI subjects at risk of AD. While there have been many studies targeting at the latter period (conversion of MCI to AD), few studies involved prediction of conversion in the earlier period (from NC to MCI), which might present better intervention effect for the subjects at risk of progression.

To differentiate the target subjects at baseline, most studies used separate MRI features as predictors [9–12], while some others attempted to combine multiple MRI features in the form of a single severity index from machine learning [8, 13, 14]. In this study, we applied such a severity index, i.e. the AD resemblance atrophy index (AD-RAI), and tested its ability to identify normal subjects who converted to MCI and MCI subjects who developed AD over a two-year period. Also, we explored the relative merits of this index (which implies complex spatial atrophy pattern of multiple brain regions) over single MRI features (i.e. the regional volumes of individual AD-related structures) for differentiation between converters and non-converters, through group comparison of baseline measures and searching the optimal cutoff (threshold) of baseline measures in logistic regressions. In addition, we measured the longitudinal trajectory of the volumetric differences between converters and non-converters to evaluate the additional value of short-term follow-up for the prediction of progression to MCI or AD at the last visit spanning two years apart.

## RESULTS

**Figure 1:**
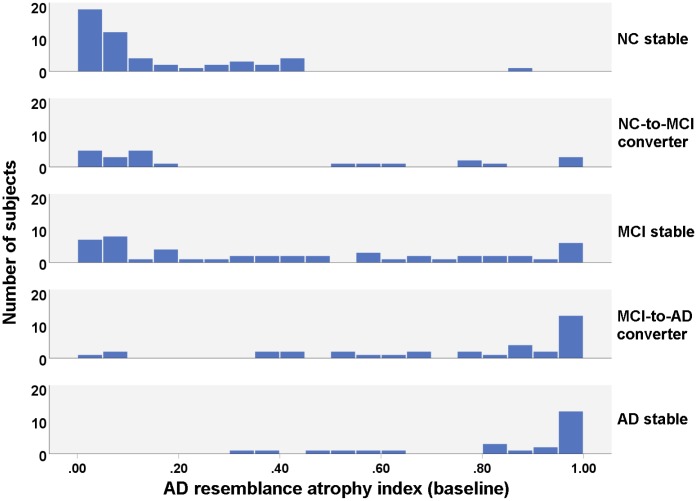
**Histogram of baseline AD resemblance atrophy index for different groups.**

The subjects of different groups were matched in age, gender and education level (Table 1). The level of CSF biomarkers (Aβ_42_, t-tau and p-tau_181_), PET-based biomarker (average cortical uptake of Florbetapir) and cognitive measures (MMSE, MoCA and its subscores) were significantly different among the groups (Table 1, Supplementary Table 2). The AD resemblance atrophy indexes of the five groups were significantly different (p<0.001) at any timepoint over the two years, indicating the differentiative ability of this atrophy index for different diagnostic status of the subjects (Figure 1). However, the change of AD-RAI over the two years was not significantly different among the groups (p=0.175).

**Table 1:** Characteristics of the subjects.

	**NCs (n=50)**	NCc (n=23)*	**MCIs (n=50)^**	**MCIc (n=35)**	**ADs (n=25)^#^**	***p***
Education (years), mean (SD)	16.46 (2.34)	16.04 (2.65)	15.92 (2.86)	16.25 (2.59)	15.48 (2.51)	0.598
Male (n (%))	30 (60%)	9 (39.10%)	30 (60%)	18 (51.40%)	14 (56%)	0.479
Baseline age (years), mean (SD)	73.3 (6.11)	74.4 (6.76)	74.7 (7.51)	73.4 (5.68)	73.6 (9.81)	0.839
CSF Aβ_42_ (pg/ml), mean (SD)						
Baseline	199.14 (51.39)	174.25 (46.07)	174.29 (45.78)	137.61 (25.29)	132.94 (41.88)	<0.001
24 months	190.63 (52.89)	169.00 (60.45)	174.03 (45.74)	129.94 (32.31)	113.59 (14.09)	<0.001
CSF t-tau (pg/ml), mean (SD)						
Baseline	75.23 (41.39)	71.09 (34.13)	81.85 (46.63)	143.20 (61.42)	138.83 (53.13)	<0.001
24 months	77.69 (54.67)	83.47 (46.83)	87.46 (55.34)	156.16 (85.28)	142.18 (59.44)	<0.001
CSF p-tau_181_ (pg/ml), mean (SD)						
Baseline	34.56 (15.85)	39.16 (25.00)	41.19 (23.75)	64.34 (28.49)	69.65 (35.15)	<0.001
24 months	44.40 (33.50)	41.95 (17.14)	51.97 (29.88)	66.40 (34.75)	74.62 (22.70)	0.025
Cortical SUVR†, mean (SD)						
Baseline	1.08 (0.14)	1.19 (0.22)	1.19 (0.20)	1.43 (0.19)	1.40 (0.21)	<0.001
24 months	1.10 (0.17)	1.16 (0.22)	1.23 (0.23)	1.42 (0.19)	1.41 (0.23)	<0.001
MMSE, mean (SD)						
Baseline	28.98 (1.20)	29.08 (1.04)	27.9 (1.65)	26.60 (1.76)	22.68 (2.05)	<0.001
6 months	28.88 (1.45)	29.00 (0.94)	27.25 (1.85)	25.40 (2.10)	22.00 (3.01)	<0.001
12 months	28.60 (1.41)	28.28 (1.67)	27.47 (2.08)	25.49 (2.51)	21.44 (3.99)	<0.001
24 months	29.10 (1.23)	28.39 (1.49)	27.16 (2.26)	22.97 (3.29)	18.20 (4.58)	<0.001
AD-RAI, mean (SD)						
Baseline	0.140 (0.170)	0.355 (0.358)	0.430 (0.349)	0.741 (0.292)	0.837 (0.218)	<0.001
6 months	0.191 (0.248)	0.292 (0.302)	0.435 (0.347)	0.759 (0.293)	0.849 (0.201)	<0.001
12 months	0.192 (0.238)	0.328 (0.348)	0.470 (0.341)	0.775 (0.276)	0.895 (0.145)	<0.001
24 months	0.167 (0.205)	0.449 (0.372)	0.522 (0.341)	0.836 (0.235)	0.911 (0.161)	<0.001
Change over 24 months	0.026 (0.070)	0.092 (0.245)	0.091 (0.171)	0.094 (0.149)	0.074 (0.119)	0.175

* Follow-up data was missing for six NCc subjects at 6 months, and five missing at 12 months; ^ Follow-up data was missing for two MCIs subjects at 6 and 12 months; ^#^ Follow-up data was missing for one ADs subject at 6 and 12 months. †Mean average cortical uptake (within frontal, anterior/posterior cingulate, lateral parietal, and lateral temporal cortex) of Florbetapir (F18-AV-45) PET with the whole cerebellum as the reference region. The available data (in terms of number of subjects) of CSF and PET biomarkers were shown in Supplementary Table 1 for each group. NCs, NC stable subjects; NCc, NC-to-MCI converters; MCIs, MCI stable subjects; MCIc, MCI-to-AD converters; ADs, AD stable subjects; SUVR, standard uptake value ratio. AD-RAI, AD resemblance atrophy index.

In the partial correlation analyses, baseline AD-RAIs were significantly associated with CSF-based Aβ_42_, t-tau and p-tau_181_ at baseline (p<0.001) and two years (p<0.05), where the associations with CSF-Aβ_42_ were stronger (Table 2). There were even stronger associations between baseline AD-RAI and average cortical uptake of Florbetapir at baseline (R=0.495, p<0.001) and two years (R=0.480, p<0.001). Of note, the associations of baseline AD-RAI with the change of these biological markers were not evaluated due to the severe data missing of CSF-based biomarkers at two years (Supplementary Table 1) and the nonsignificant change of PET-based biomarker over the two years. In addition, baseline AD-RAIs were significantly associated with MMSE and MoCA at baseline and two years as well as the deterioration of these two scores over the two years (p<0.001) (Table 3). Specifically, baseline AD-RAIs were also positively associated with the decline of domain scores of MoCA over the two years, including memory (p<0.001), visuospatial function (p=0.021), language (p<0.001) and attention (p<0.001).

**Table 2:** Correlation of AD resemblance atrophy index and biomarkers.

**Biomarkers**		**AD resemblance atrophy index (baseline)**	
		**Partial correlation**	**p-value**
CSF Aβ_42_	Baseline	-0.453	1.13E-09
	24 months	-0.472	1.18E-06
CSF t-tau	Baseline	0.371	1.23E-06
	24 months	0.324	0.001
CSF p-tau_181_	Baseline	0.380	5.02E-07
	24 months	0.254	0.013
Mean cortical SUVR*	Baseline	0.495	1.05E-12
	24 months	0.480	7.47E-11

Pearson partial correlation analyses were performed with age and gender as covariates. The data (number of subjects) available for analyses was shown in Supplementary Table 1. *Mean average cortical uptake (within frontal, anterior/posterior cingulate, lateral parietal, and lateral temporal cortex) of Florbetapir (F18-AV-45) PET with the whole cerebellum as the reference region. SUVR, standard uptake value ratio.

### Difference of baseline brain volumetry between converters and non-converters

#### NC-to-MCI converter (NCc) vs. NC stable (NCs)

**Figure 2:**
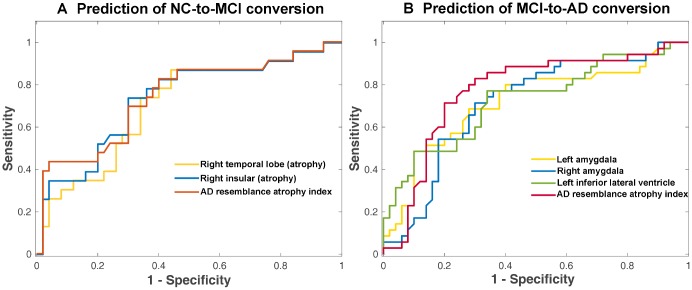
**ROC curve of prediction of conversion to MCI in NC subjects (A) and conversion to AD in MCI subjects (B) from logistic regression.** Only the brain volumetric measures that achieved an AUC of >0.7 with the optimized cutoff (as shown in Table 5 and Table 7) were displayed here.

Comparing NCs and NCc converters, the converters had presented higher AD-RAI (p=0.004) as well as regional brain volume loss as identified by single regions (right temporal lobe, left insular and right insular, p<0.05) at baseline, as shown in Table 4. We further searched for the best cutoff of these four measures in logistic regressions and measured the AUC of the logistic regression models with the best cutoff. The left insular atrophy was not predictive of the conversion status with any cutoff (p>0.05) and therefore was not shown in Table 5. Among the remaining volumetric measures (also shown in Figure 2A with ROC curves), the AD-RAI presented the highest AUC (0.740) and odds ratio (OR=26.35, p=0.003) for prediction of conversion status (NCc vs. NCs), with 0.5 as the best cutoff value.

**Table 3:** Partial correlation of AD resemblance atrophy index and neuropsychological tests.

**Neuropsychological tests**		**AD resemblance atrophy index (baseline)**	
		**Partial correlation**	**p-value**
MMSE	Baseline	-0.570	8.41E-16
	24 months	-0.637	2.01E-20
	Decline in 2 years	0.461	1.02E-10
MoCA	Baseline	-0.394	1.39E-07
	24 months	-0.538	6.16E-14
	Decline in 2 years	0.389	8.70E-08
MoCA-memory	Baseline	-0.289	1.52E-04
	24 months	-0.508	2.55E-12
	Decline in 2 years	0.323	1.13E-05
MoCA-visuospatial	Baseline	-0.210	6.51E-03
	24 months	-0.357	2.23E-06
	Decline in 2 years	0.174	0.021
MoCA-language	Baseline	-0.124	0.110
	24 months	-0.350	3.48E-06
	Decline in 2 years	0.258	5.30E-04
MoCA-attention	Baseline	-0.260	6.82E-04
	24 months	-0.405	5.82E-08
	Decline in 2 years	0.270	2.81E-04
MoCA-executive	Baseline	-0.288	1.64E-04
	24 months	-0.376	5.45E-07
	Decline in 2 years	0.127	0.092

Pearson partial correlation analyses were performed with age, gender and education year as covariates.

**Table 4:** Comparison of baseline MMSE and brain volumetry in NC stable and NC-to-MCI converters.

**Measure**	**NC stable (n=50)**	**NC-to-MCI converter (n=23)**	***p***
MMSE	28.98 (1.20)	29.08 (1.04)	0.355
AD resemblance atrophy index*	0.141 (0.171)	0.356 (0.365)	0.004
Brain parenchyma	75.284 (1.834)	74.783 (1.905)	0.668
Hippocampus L	0.218 (0.018)	0.211 (0.020)	0.287
Hippocampus R	0.225 (0.019)	0.219 (0.021)	0.532
Amygdala L	0.114 (0.011)	0.114 (0.012)	0.622
Amygdala R	0.138 (0.014)	0.132 (0.015)	0.409
Ventricular system	10.034 (1.193)	10.527 (1.320)	0.295
Lateral ventricle L	1.048 (0.440)	1.222 (0.651)	0.329
Lateral ventricle R	0.977 (0.386)	1.244 (0.720)	0.061
Inferior lateral ventricle L	0.011 (0.005)	0.016 (0.016)	0.103
Inferior lateral ventricle R	0.008 (0.004)	0.012 (0.016)	0.164
Frontal lobe (atrophy) L	41.996 (6.840)	42.204 (5.884)	0.777
Frontal lobe (atrophy) R	43.138 (6.174)	44.243 (6.851)	0.872
Occipital lobe (atrophy) L	14.936 (3.016)	15.089 (4.250)	0.951
Occipital lobe (atrophy) R	11.335 (2.261)	11.423 (3.283)	0.891
Temporal lobe (atrophy) L	25.890 (4.334)	28.204 (6.327)	0.144
Temporal lobe (atrophy) R*	20.502 (3.596)	23.639 (4.218)	0.002
Parietal lobe (atrophy) L	45.210 (9.082)	41.009 (12.357)	0.058
Parietal lobe (atrophy) R	41.352 (8.441)	39.057 (10.553)	0.120
Cingulate lobe (atrophy) L	10.216 (3.290)	11.156(5.656)	0.273
Cingulate lobe (atrophy) R	17.298 (5.549)	18.260 (8.291)	0.346
Insular (atrophy) L*	21.382 (6.552)	26.009 (11.128)	0.047
Insular (atrophy) R*	14.210 (4.932)	18.239 (7.948)	0.017
White matter hyperintensity	0.572 (0.776)	0.615 (0.503)	0.839

The comparison was performed with ANCOVA with age and gender as covariates. The mean and SD of AD resemblance atrophy index and single regional volumetric measures in both groups are provided. *Measures that were significantly different between NC stable and NC-to-MCI converters (p<0.05). L, left; R, right.

**Table 5:** Differentiation in NC stable and NC-to-MCI converters using AD atrophy index and single regional volumetric measures.

**Measure**	**Cutoff**	**Odds ratio (95% CI) ^**	***p***	**AUC (95% CI)**
AD resemblance atrophy index	0.4	4.75 (1.32, 17.08)	0.017	0.714 (0.584, 0.844)
	0.5*	**26.35 (2.96, 234.77)**	**0.003**	**0.740 (0.612, 0.868)**
	0.6	16.75 (1.83, 153.20)	0.013	0.718 (0.589, 0.847)
	0.7	13.53 (1.45, 126.53)	0.022	0.710 (0.580, 0.839)
Temporal lobe (atrophy) R	50%	3.31 (1.07, 10.19)	0.037	0.696 (0.571, 0.822)
	75%	3.44 (1.04, 11.39)	0.043	0.692 (0.559, 0.824)
	85%*	4.88 (1.13, 21.08)	0.033	0.703 (0.574, 0.832)
Insular (atrophy) R	75%	5.03 (1.49, 16.97)	0.009	0.715 (0.584, 0.847)
	80%	5.05 (1.39, 18.42)	0.014	0.722 (0.592, 0.852)
	85%	4.96 (1.17, 20.93)	0.029	0.715 (0.584, 0.845)
	90%*	19.59 (1.91, 201.75)	0.012	0.733 (0.606, 0.860)

The AD resemblance atrophy index and the single regional volumetric measures were dichotomized with cutoffs to evaluate their performance in differentiating NC stable and NC-to-MCI converters using logistic regression. Age and gender were covaried out. Only the measures that were significantly different between NC stable and NC-to-MCI converters were tested (as labeled in Table 4) and only the measures with a cutoff that achieved p<0.05 in logistic regression are shown here. The searching range of cutoff is 0.1~0.9 (real value) in increments of 0.1 for AD resemblance atrophy index and typical percentiles (50%, 75%, 80%, 85%, 90%) for individual lobar atrophy measures. *The optimal cutoff value for a specific measure in logistic regression. R, right.

### MCI-to-AD converter (MCIc) vs. MCI stable (MCIs)

There were more brain volumetric measures that had presented baseline differences between MCIc and MCIs (p<0.05) than NCc vs. NCs (Table 6). These baseline volumetric measures that provided clues for future progression included AD-RAI, volume ratios of bilateral hippocampus, bilateral amygdala and left inferior lateral ventricle, and atrophy degree of left occipital lobe, bilateral temporal lobe and right insular (Table 6). Among these volumetric measures, the AD-RAI with a cutoff of 0.5 achieved the highest AUC (0.771) with an odds ratio of 8.91 (p<0.001) (Table 7). The volumetric measures that achieved an AUC of >0.7 were also shown in Figure 2B with ROC curves plotted.

**Table 6:** Comparison of baseline MMSE and brain volumetry in MCI stable and MCI-to-AD converters.

**Measure**	**MCI stable (n=50)**	**MCI-to-AD converter (n=35)**	***p***
MMSE	27.9 (1.65)	26.60 (1.76)	0.001
AD resemblance atrophy index*	0.430 (0.349)	0.741 (0.292)	<0.001
Brain parenchyma	73.850 (2.321)	73.580 (2.158)	0.170
Hippocampus L*	0.203 (0.020)	0.191 (0.027)	0.024
Hippocampus R*	0.211 (0.021)	0.201 (0.024)	0.040
Amygdala L*	0.106 (0.014)	0.096 (0.015)	0.003
Amygdala R*	0.127 (0.018)	0.116 (0.016)	0.003
Ventricular system	10.617 (1.679)	10.687 (1.310)	0.356
Lateral ventricle L	1.307 (0.767)	1.342 (0.543)	0.462
Lateral ventricle R	1.200 (0.682)	1.235 (0.577)	0.385
Inferior lateral ventricle L*	0.013 (0.010)	0.017 (0.008)	0.010
Inferior lateral ventricle R	0.010 (0.006)	0.011 (0.006)	0.245
Frontal lobe (atrophy) L	45.976 (7.464)	44.480 (7.543)	0.461
Frontal lobe (atrophy) R	46.416 (7.138)	44.814 (6.451)	0.454
Occipital lobe (atrophy) L*	16.478 (3.798)	18.179 (4.848)	0.034
Occipital lobe (atrophy) R	12.749 (3.958)	13.770 (4.766)	0.150
Temporal lobe (atrophy) L*	29.394 (6.493)	32.429 (7.682)	0.009
Temporal lobe (atrophy) R*	23.052 (4.996)	25.346 (5.692)	0.005
Parietal lobe (atrophy) L	47.536 (10.466)	48.683 (10.114)	0.629
Parietal lobe (atrophy) R	42.768 (8.664)	45.514 (9.116)	0.132
Cingulate lobe (atrophy) L	11.896 (4.728)	12.928 (4.913)	0.117
Cingulate lobe (atrophy) R	18.646 (6.619)	20.511 (7.003)	0.067
Insular (atrophy) L	25.652 (10.621)	28.041 (11.253)	0.093
Insular (atrophy) R*	16.277 (6.242)	17.849 (6.433)	0.043
White matter hyperintensity	0.786 (0.667)	0.524 (0.418)	0.080

The comparison was performed with ANCOVA with age and gender as covariates. The mean and SD of AD resemblance atrophy index and single regional volumetric measures in both groups are provided. *Measures that were significantly different between MCI stable and MCI-to-AD converters (p<0.05). L, left; R, right.

**Table 7:** Differentiation in MCI stable and MCI-to-AD converters using AD atrophy index and single regional volumetric measures.

**Measure**	**Cutoff**	**Odds ratio (95% CI) ^**	***p***	**AUC (95% CI)**
AD resemblance atrophy index	0.1	4.62 (1.21, 17.69)	0.026	0.684 (0.568, 0.800)
	0.2	7.07 (1.87, 26.69)	0.004	0.722 (0.610, 0.834)
	0.3	8.55 (2.27, 32.13)	0.001	0.745 (0.635, 0.854)
	0.4	7.56 (2.37, 24.13)	0.001	0.750 (0.641, 0.858)
	0.5*	**8.91 (2.81, 28.31)**	**<0.001**	**0.771 (0.664, 0.877)**
	0.6	6.12 (2.20, 16.99)	0.001	0.744 (0.636, 0.852)
	0.7	4.91 (1.88, 12.86)	0.001	0.719 (0.608, 0.831)
	0.8	5.39 (2.01, 14.43)	0.001	0.720 (0.609, 0.831)
	0.9	5.56 (1.86, 16.61)	0.002	0.703 (0.590, 0.816)
Hippocampus L	50%	2.90 (1.17, 7.18)	0.021	0.660 (0.539, 0.782)
	25%*	4.96 (1.65, 14.92)	0.004	0.679 (0.559, 0.798)
	20%	4.90 (1.50, 16.02)	0.009	0.667 (0.546, 0.788)
	15%	6.60 (1.53, 28.38)	0.011	0.677 (0.558, 0.797)
Hippocampus R	20%*	3.43 (1.10, 10.69)	0.033	0.656 (0.534, 0.777)
	10%	9.27 (1.01, 84.76)	0.049	0.642 (0.521, 0.763)
Amygdala L	50%	3.30 (1.31, 8.33)	0.012	0.661 (0.543, 0.780)
	25%*	7.33 (2.33, 23.02)	0.001	0.720 (0.605, 0.834)
	20%	4.64 (1.45, 14.86)	0.010	0.674 (0.555, 0.793)
	15%	5.61 (1.36, 23.12)	0.017	0.666 (0.544, 0.787)
Amygdala R	50%*	4.96 (1.91, 12.89)	0.001	0.712 (0.599, 0.825)
	25%	4.73 (1.60, 14.00)	0.005	0.694 (0.578, 0.809)
Inferior lateral ventricle L	50%*	5.29 (1.90, 14.69)	0.001	0.726 (0.614, 0.838)
	75%	7.23 (2.29, 22.87)	0.001	0.725 (0.614, 0.835)
	80%	4.03 (1.27, 12.74)	0.018	0.661 (0.543, 0.779)
Temporal lobe (atrophy) L	50%	3.26 (1.21, 8.84)	0.020	0.663 (0.545, 0.781)
	75%*	6.01 (1.87, 19.33)	0.003	0.689 (0.574, 0.805)
	80%	5.04 (1.48, 17.18)	0.010	0.662 (0.543, 0.780)
	85%	5.28 (1.30, 21.51)	0.020	0.656 (0.538, 0.774)
Temporal lobe (atrophy) R	50%	2.88 (1.10, 8.20)	0.048	0.651 (0.532, 0.770)
	75%*	4.54 (1.48, 13.91)	0.008	0.678 (0.561, 0.796)
	80%	3.22 (1.02, 10.16)	0.046	0.641 (0.520, 0.762)
Insular (atrophy) R	50%*	4.27 (1.49, 12.19)	0.007	0.688 (0.571, 0.806)

The AD resemblance atrophy index and the single regional volumetric measures were dichotomized with cutoffs to evaluate their performance in differentiating MCI stable and MCI-to-AD converters using logistic regression. Age and gender were covaried out. Only the measures that were significantly different between MCI stable and MCI-to-AD converters were tested (as labeled in Table 6) and only the measures with a cutoff that achieved p<0.05 in logistic regression are shown here. The searching range of cutoff is 0.1~0.9 (real value) in increments of 0.1 for AD resemblance atrophy index, (50^th^, 75^th^, 80^th^, 85^th^, 90^th^) percentiles for individual lobar atrophy measures and ventricle measures, and (10^th^, 15^th^, 20^th^, 25^th^, 50^th^) percentiles for subcortical measures. *The optimal cutoff value for a specific measure in logistic regression.

### Longitudinal volumetric changes of converters and non-converters

To explore whether the brain volumetric measures that presented difference between converters and non-converters at baseline would also have differed longitudinal trajectory, we performed linear mixed effect model analyses, where the brain volumetric data at 6, 12 and 24 months were additionally used.

### NC-to-MCI converter vs. NC stable

**Figure 3:**
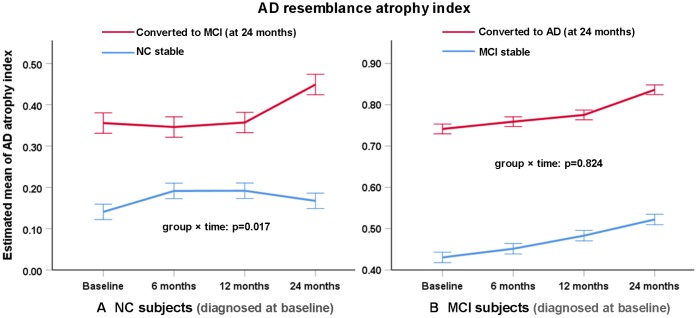
Change of AD resemblance atrophy index of (**A**) NC and (**B**) MCI subjects as diagnosed at baseline over two years. Figure shows estimated mean change in AD atrophy index from baseline until 6, 12 and 24 months (higher scores suggest more severe atrophy). Error bars are standard errors. Mixed-model repeated-measures analyses were used to assess between-group differences (group × time interaction) in changes from baseline to 24 months.

Regarding the AD-RAI, it had slight but significant longitudinal increase (p=0.017) in NCc subjects compared with NCs subjects (especially during 12~24 months), and it well differentiated the two groups at any timepoint (Figure 3A). The other volumetric measures did not present a significant group × time interaction (p>0.05).

### MCI-to-AD converter vs. MCI stable

**Figure 4:**
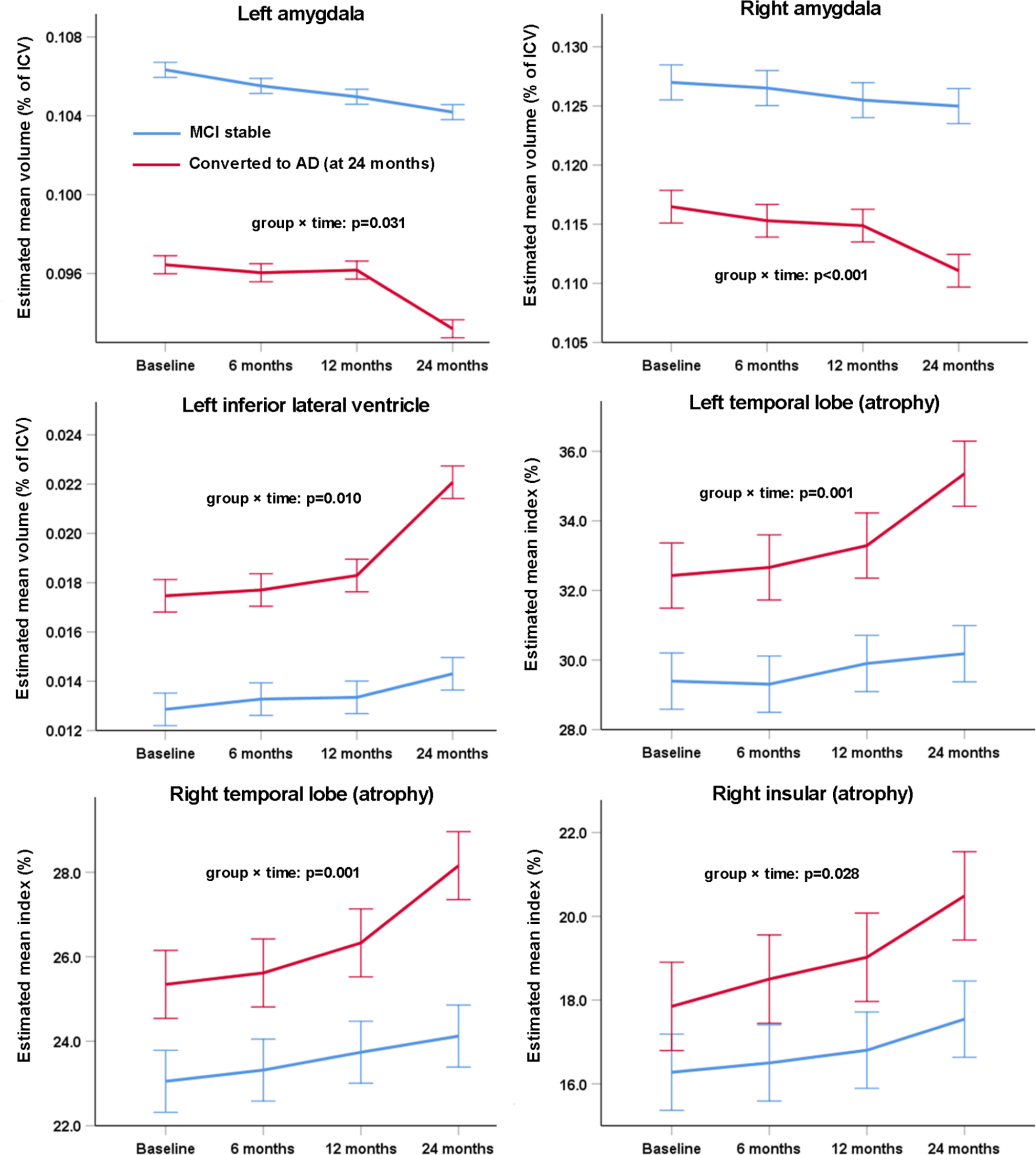
**Change of individual volumetric measures of MCI subjects as diagnosed at baseline over two years.** Error bars are standard errors. Mixed-model repeated-measures analyses were used to assess between-group differences (group × time interaction) in changes from baseline to 24 months. Only the measures that were significantly different between MCI stable and MCI-to-AD converters were tested (as labeled in Table 6) and only the measures with a significant group × time interaction in the subsequent analyses are shown here.

Although the AD-RAI (based on brain volumetry of multiple regions) differentiated MCIc vs. MCIs at any timepoint over the two years (Figure 3B), there were no significant group × time interaction (p=0.824), indicating that the longitudinal changes in AD-RAI of the two groups were similar. In contrast, there were many regional volumetric measures that presented significant group × time interactions (Figure 4), such as left amygdala (p=0.031), right amygdala (p<0.001), left inferior lateral ventricle (p=0.010), left temporal lobe (p=0.001), right temporal lobe (p=0.001) and right insular (p=0.028). The difference of longitudinal changes between groups was only obvious in the period of 12~24 months.

### Representative cases of converters and non-converters

**Figure 5:**
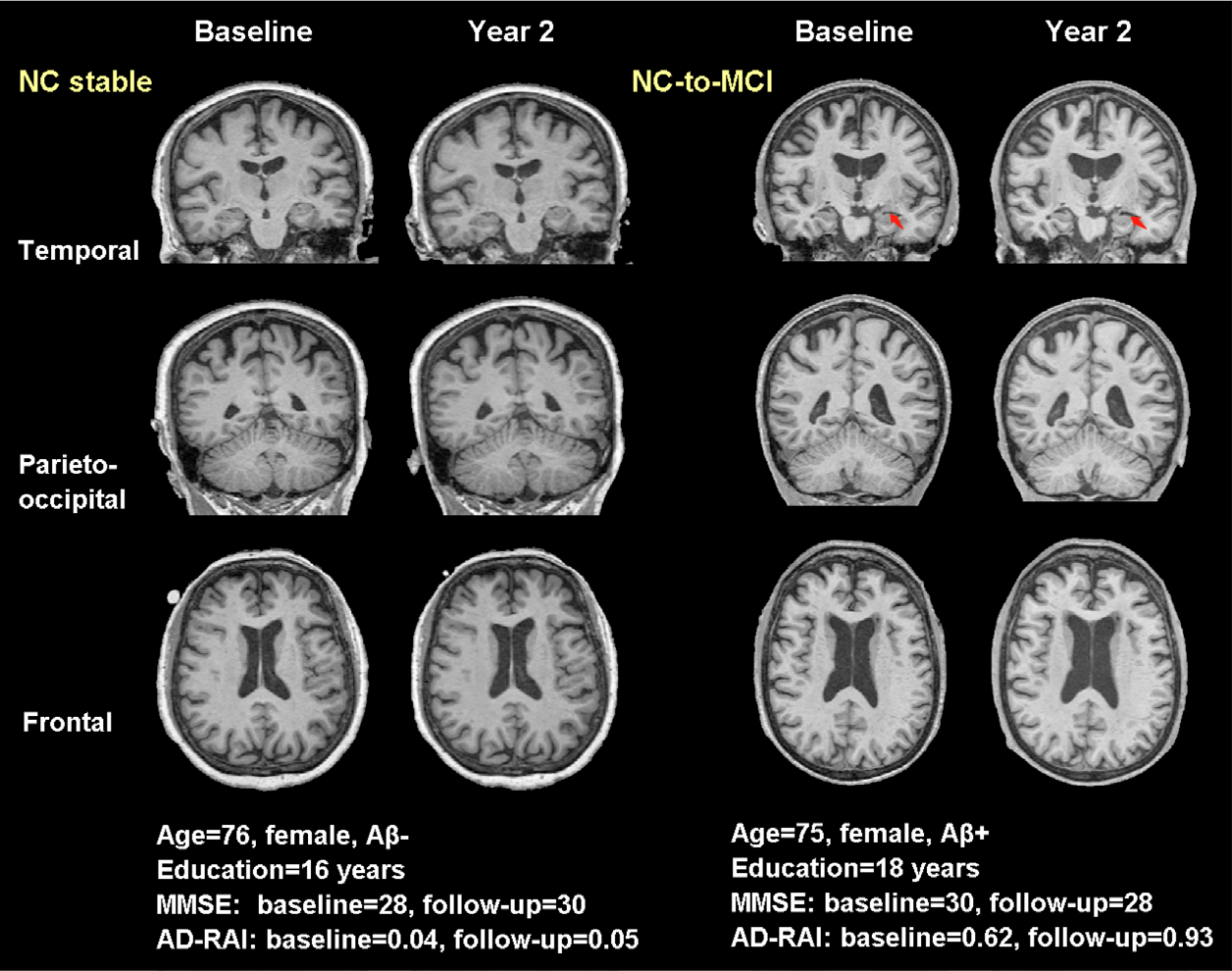
**Typical real cases of NC stable and NC-to-MCI subjects.** T1-weighted (T1W) images at baseline and two years were shown for the two typical cases in temporal, parieto-occipital and frontal view. Red arrows pointed to the region with significant atrophy by comparing the T1W images of the same subject over two years. The typical case of NC stable did not present atrophy while the case of NC-to-MCI showed increased width of left choroid fissure and temporal horn (temporal view). Aβ-: CSF-based Aβ_42_ >192 pg/ml at baseline and 2 years; Aβ+: CSF-based Aβ_42_ <192 pg/ml at baseline and 2 years; AD-RAI: AD resemblance atrophy index.

**Figure 6:**
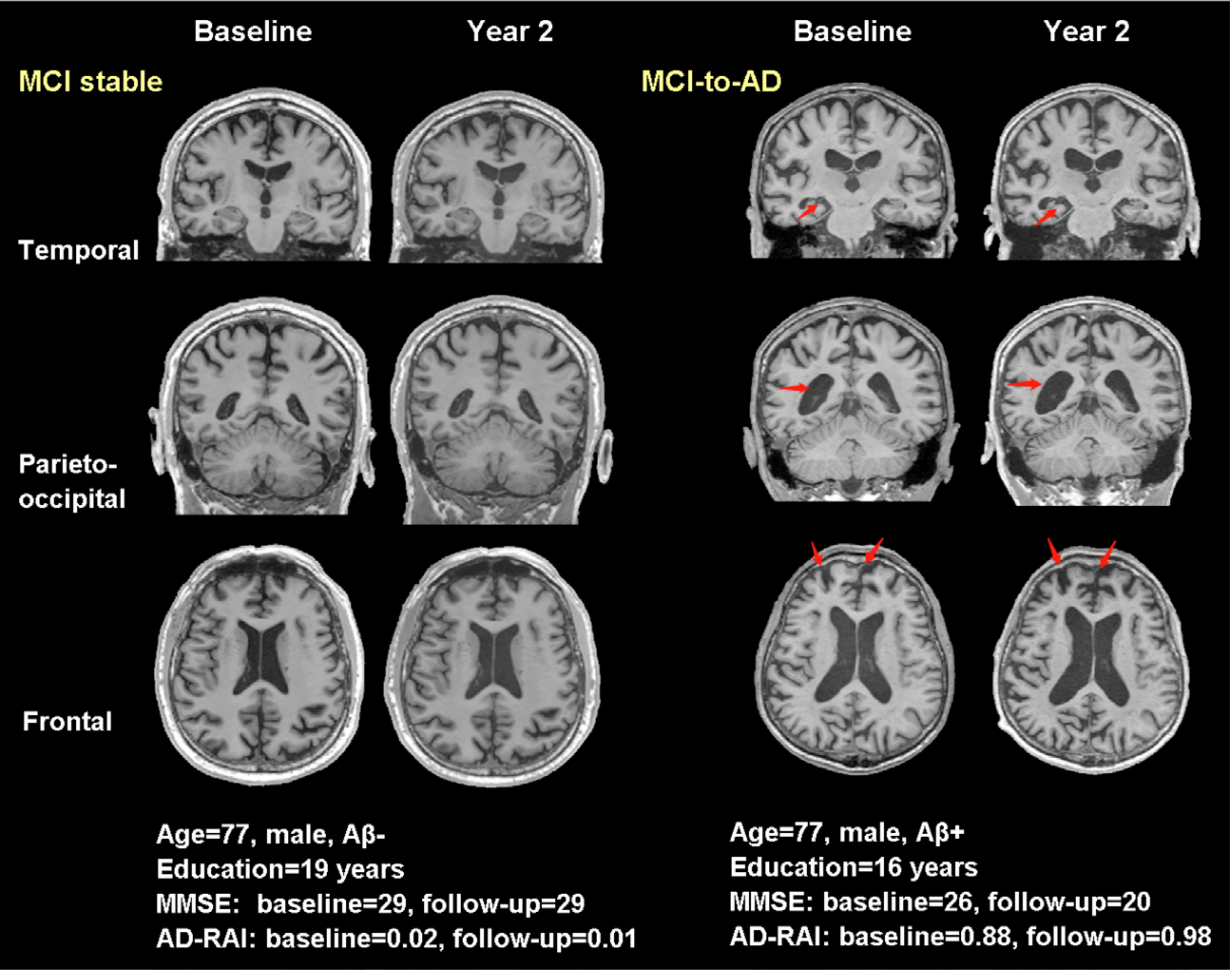
**Typical real cases of MCI stable and MCI-to-AD subjects.** T1-weighted (T1W) images at baseline and two years were shown for the two typical cases in temporal, parieto-occipital and frontal view. Red arrows pointed to the regions with significant atrophy by comparing the T1W images of the same subject over two years. The typical case of MCI stable did not present progressed atrophy during the two years, while the case of MCI-to-AD showed increased width of right choroid fissure and temporal horn (temporal view), enlargement of lateral ventricle (parieto-occipital view) and increased frontal lobe atrophy (frontal view). Aβ-: CSF-based Aβ_42_ >192 pg/ml at baseline and 2 years; Aβ+: CSF-based Aβ_42_ <192 pg/ml at baseline and 2 years; AD-RAI: AD resemblance atrophy index.

Also, we complemented with four typical real cases in Figure 5 (NCs vs. NCc) and Figure 6 (MCIs vs. MCIc) to illustrate the effect of AD-RAI in evaluation of disease progression (the characteristics of these subjects were provided on the figures). While the sample case of NCs (baseline AD-RAI=0.04) did not present significant atrophy over the two years, the sample case of NCc (baseline AD-RAI=0.62) showed increased width of left choroid fissure and temporal horn (Figure 5). Similarly, the sample case of MCIs (baseline AD-RAI=0.02) did not present progressed atrophy during the two years, while the sample case of MCIc (baseline AD-RAI=0.88) showed increased width of right choroid fissure and temporal horn, enlargement of lateral ventricle, and increased frontal lobe atrophy (Figure 6).

## DISCUSSION

In this study, we compared a synthetic atrophy index (AD-RAI) derived from multiple brain regions with single regional volumetric measures in differentiating at-risk subjects two years before progression from NC to MCI and from MCI to AD. The findings of this study confirm the effectiveness of using baseline AD-RAI in differentiating subjects at risk of conversion to MCI (from NC) and AD (from MCI) in a two-year follow-up, although choosing the optimal cutoff values of this index for specific individual-level differentiation tasks remains a challenge.

Regarding the subjects presented as cognitive-intact (NC) at baseline, the NCc subjects had no significant difference in baseline MMSE compared with NCs subjects (Table 4). In contrast, AD-RAI and several individual regional volumetric measures (i.e. right temporal lobe, left insular and right insular) showed significant difference between these two groups (Table 4). These results generally corroborated with the previous studies, as greater right temporal lobe atrophy has been reported in normal subjects years before progression to AD [17], and insular atrophy has been identified in MCI patients [18]. However, hippocampus atrophy, which was reported as an early biomarker of AD conversion for NC subjects [15, 19], was not found in the comparison of NCc vs. NCs subjects at baseline in our study. It may result from the shorter duration of follow-up (two years) in this study compared with those targeting at NCc subjects over a period of more than five years, and that the NCc subjects may not necessarily present AD-like atrophy pattern as not all MCI patients would convert to AD. In the subsequent logistic regressions, the AD-RAI measured at baseline with a cutoff of 0.5 performed the best for differentiating NCc vs. NCs subjects spanning two years apart (Table 5). Previous studies have reported the use of such an atrophy severity index of AD (based on complex AD-like atrophy pattern of multiple regions) in differentiating MCIc from MCIs subjects [8, 13, 14]. For the first time, we demonstrated that such an index could also differentiate normal subjects at risk of conversion to MCI over a two-year period (as illustrated in Figure 5 with real cases).

Regarding the subjects diagnosed as MCI at baseline, the MCIc patients had already presented lower baseline MMSE than MCIs patients, and there were many individual regional volumetric measures in additional to AD-RAI that presented significant difference between the converters vs. non-converters (Table 6). It indicated that there has been widespread greater brain atrophy in multiple brain regions in MCIc than MCIs patients [14], as illustrated with real cases in Figure 6. Among the regions that presented greater atrophy in MCIc (Table 6), hippocampus, amygdala, temporal lobe and insular have been frequently reported as early biomarkers in differentiating converters vs. non-converters from MCIc [12, 18, 20, 21]. The performance of occipital lobe atrophy in differentiating MCIc from MCIs patients was also reported in a previous study (AUC=0.59) [21]. The enlargement of inferior lateral ventricle has been identified in MCI and AD patients [22, 23] and used in multivariate analysis to differentiate MCIc patients [24]. In the subsequent logistic regressions with cutoff searching, most of these individual regional volumetric measures well differentiated MCIc from MCIs patients, but they still presented inferior performance compared with AD-RAI (with 0.5 as the optimal cutoff) as shown in Table 7 and Figure 2B. Compared with several previous studies that also investigated a single synthetic atrophy index (based on complex AD-like atrophy pattern of multiple brain regions) at baseline for differentiation of at-risk subjects of MCI-to-AD conversion [8, 13, 14], we achieved similar performance (AUC: 0.771 in this study and 0.675~0.770 in previous studies).

Of note, the optimal threshold of AD-RAI for differentiation of converters and non-converters should be explained with caution. In this study, we found that the optimal cutoff of AD-RAI for differentiation of NCc vs. NCs and MCIc vs. MCIs was the same (i.e. 0.5). While this may indicate similar baseline atrophy difference of converters in NC and MCI, further validations are still needed, because the differentiation performance with other cutoffs (e.g. 0.4 for NCc vs. NCs, and 0.6 for MCIc vs. MCIs) did not differ a lot from 0.5. In this regard, it remains a challenge to determine optimal cutoff points for such index [14], where larger sample size is needed to test the reliability of differentiations with specific cutoffs. Finally, the WMH volume measured at baseline was not significantly different in both comparisons of NCc vs. NCs and MCIc and MCIs, indicating that the vascular factors might not have significant impact on the differentiation of the converters vs. non-converters.

Also, we compared the longitudinal trajectories of these atrophy measures in converters and non-converters. The NCc subjects presented more rapid increase of AD-RAI than NCs subjects (during the second year), while MCIc patients showed similar growth rate of AD-RAI with MCIs patients. It may indicate that the potential of including AD-RAI of short-term follow-up(s) for a better prediction of progression from NC to MCI. However, as NCc showed more rapid brain atrophy than NCs only at the last visit (two years), the additional contribution of AD-RAI at short-term follow-ups for long-term NC-to-MCI conversion still needs to be validated with larger sample size and more intensive follow-ups. Of note, there were many individual regional volumetric measures that showed greater atrophy rate in MCIc than MCIs patients (Figure 4). In fact, baseline AD-RAI has outperformed these individual regional measures in differentiating MCIc from MCIs, and the more rapid atrophy of these measures generally occurred at the last visit. Therefore, the contribution of evaluating these individual regional volumetric measures to prediction of MCI-to-AD conversion should be further validated in the future as well.

In addition to the analyses within specific groups (NC or MCI as diagnosed at baseline), we also found significant associations between AD-RAI and well-established AD biomarkers [25] at baseline (such as Aβ quantified from CSF or F18-AV-45 PET and tau from CSF) in the entire cohort (Table 2). These results indicated the consistency of MRI-based volumetry with early AD biological markers, and this non-invasive MRI-based atrophy index (AD-RAI) might facilitate early screening of general population for the risk of AD-related disease progression. The associations of AD-RAI with other AD biomarkers (e.g. retinal conditions [26]) may be evaluated in the future when multiple types of AD biomarkers are available. Furthermore, the AD-RAI presented significant associations with deterioration of global cognition and domain cognitive function (Table 3, Supplementary Table 2), which corroborated with our positive findings regarding the potential of AD-RAI in predicting NC-to-MCI conversion and MCI-to-AD conversion. As the domain cognitive functions were only assessed with the components of MoCA, future work should apply a more detailed battery of neuropsychological assessments to explore the potential of AD-RAI in detecting the population at risk of cognitive decline in specific domains.

There are several limitations to this study that should be considered. Firstly, the sample size of the study cohort is relatively small (especially for NCc group), which makes it difficult to perform a more comprehensive searching of the cutoffs for both AD-RAI and volumetric measures of individual regions, because at least a number of subjects need to be allocated to the smaller dichotomized group. Therefore, further validations are needed to test whether the findings of this study (e.g. optimal cutoff of baseline AD-RAI for future conversion to MCI/AD) can be generalizable to a larger cohort. In addition, although this study aimed to measure separate periods of AD progression (NC-to-MCI, and MCI-to-AD), the follow-up duration (2 years) is still short to capture sufficient brain volumetric changes. There might be some subjects that would have progression soon after the last visit but were still diagnosed as NCs or MCIs based on the observations within two years. Also, some of the imaging data of intermediate visits (at 6 and 12 months) were missing, and there was one subject that had reversion from MCI to NC during the intermediate visits. However, the longitudinal trajectory analyses that involved intermediate visits were performed with mixed effect model which is resistant to missing data. The only one subject with short-term reversion from MCI to NC returned to MCI at the last visit and the disease progressions of remaining subjects did not reverse in the two years. Finally, this study aimed to test the ability of brain volumetric measures (based on structural MRI) in identifying the risk of conversion to MCI/AD, and no other biomarkers were used for the predictions. As different biomarkers (e.g. PET, CSF and neuropsychological assessments) provide complementary information and presented better prediction of conversion [27–29], further efforts should be made to combine these features in a single synthetic AD risk index as an easy-to-use tool for individual-level diagnosis.

In conclusion, this study confirmed the potential of using synthetic atrophy index that combines brain volumetric measures of multiple regions in early differentiation of subjects at risk of conversion from NC to MCI and from MCI to AD at an individual-level. Future efforts should aim to identify a reliable cutoff of this index in specific differentiation tasks, where a longer duration of follow-up and larger sample size would be preferred. The additional contribution of short-term follow-ups of this index for prediction of conversion also needs to be validated with a larger cohort.

## MATERIALS AND METHODS

### Subjects

All data used in this study was obtained from the Alzheimer’s Disease Neuroimaging Initiative (ADNI) database (http://adni.loni.usc.edu), which was launched in 2003 as a public-private partnership. The primary goal of ADNI has been to test whether serial magnetic resonance imaging (MRI), positron emission tomography (PET), other biological markers, and clinical and neuropsychological assessment can be combined to measure the progression of mild cognitive impairment (MCI) and early Alzheimer’s disease (AD) [30].

Data used in this work included subjects from the Alzheimer's Disease Neuroimaging Initiative phase 2 (ADNI-2) who had both baseline and follow-up MRI data and diagnostic information spanning two years apart. To map a more comprehensive trajectory of the changes during the two years, we also included the follow-up data (MRI scans and diagnostic information) at 6 and 12 months of these subjects if available. All MRI scans were checked for quality control, and those with common artifacts or structural abnormalities were excluded from the dataset [31]. Neuropsychological test scores were also obtained such as Mini-Mental State Examination (MMSE) [32] and Montreal Cognitive Assessment (MoCA) [33]. Five cognitive domain scores of MoCA were also calculated using a method published previously [34], including memory, language, attention, executive function and visuospatial function. In addition, we downloaded the data of CSF-based biomarkers such as amyloid-β (Aβ_42_), total tau (t-tau) and phosphorylated tau (p-tau_181_), and the processed data of Florbetapir (F18-AV-45) PET in terms of average cortical uptake with the whole cerebellum as the reference region [35].

According to the diagnostic information at baseline and 24 months, each subject was assigned to one of the following groups: (1) NC-to-MCI converter, (2) MCI-to-AD converter, (3) AD stable, (4) NC stable, (5) MCI stable. Information on the change of subjects’ diagnoses were downloaded from the ADNI website (DXSUM_PDXCONV_ADNIALL.csv). For group (1) and (2), converter is defined when a subject’s diagnostic status has advanced during the two-year period. We included NC-to-MCI converter (NCc) subjects who have transitioned from NC to MCI, and MCI-to-AD converter (MCIc) subjects who transitioned from MCI to AD from baseline to 24-month follow-up examinations. For subjects in groups (3), (4) and (5), stable is defined when a subject kept his/her baseline diagnosis for the whole two-year period. Finally, we identified 23 NCc subjects, 35 MCIc subjects, 25 AD stable (ADs) subjects, 50 NC stable (NCs) subjects and 50 MCI stable (MCIs) subjects. The diagnostic information of these subjects over the 2 years were shown in Table 8.

**Table 8:** Diagnostic distribution at each visit over 2 years.

	**Baseline**	**6 months**	**12 months**	**24 months**
Cumulative	(n=183)	(n=174)	(n=175)	(n=183)
NC	73	66	63	50
MCI	85	76	73	73
AD	25	32	39	60
Missing	0	9	8	0
Conversion (compared with baseline)				
NC to MCI	0	3	5	23
MCI to AD	0	10	9	35
MCI to NC	0	1	1	0

### MRI acquisition and processing

High-resolution structural brain MRI scans were acquired using 3T MRI scanners (GE Healthcare, Philips Medical Systems, or Siemens). For T1-weighted MRI, GE scanners use inversion recovery-fast spoiled gradient recalled (IR-FSPGR) sequences and Philips and Siemens use magnetization-prepared rapid gradient echo (MP-RAGE) sequences. For T2-weighted MRI, all the scanners use Axial T2 fluid attenuated inversion recovery (FLAIR) sequence.

All the MRIs were processed using AccuBrain^®^ (BrainNow Medical Technology Limited), a cloud-based tool of automated brain volumetry that performs brain structure and tissue segmentation and quantification in a fully automatic mode. In a recent validation study based on a standard dataset from the European Alzheimer’s Disease Consortium - Alzheimer’s Disease Neuroimaging Initiative Harmonized Protocol (EADC-ADNI HarP) where manual hippocampal segmentation reference was available, AccuBrain^®^ achieved the best performance among the existing automatic brain segmentation tools [36]. In this study, we selected brain parenchyma, typical subcortical structures (bilateral hippocampus and amygdala), ventricular regions (ventricular system, lateral ventricle, inferior lateral ventricle) and lobar regions (frontal lobe, occipital lobe, temporal lobe, parietal lobe, cingulate lobe and insular) for quantification of brain volumetry, which are cognitive-relevant regions for the subsequent analysis. In detail, the subcortical regions and ventricle structures were measured with volume ratio (% of intracranial volume (ICV)), and the cortical regions were measured with atrophy degree regarding the ratio of the volume of cerebrospinal fluid (CSF) to cortical volume of a specific region [37]. To investigate the influence of small vessel disease on the outcomes of the study cohort, we also quantified the total volume of white matter hyperintensities (WMH) for each subject using AccuBrain^®^, based on an automated WMH segmentation algorithm mentioned in a previous study [38]. The WMH volumes to be compared between groups were also normalized by ICV as volume ratios (% of ICV).

In addition to the brain structural volumetry, an AD resemblance structural atrophy index (AD-RAI) was also estimated for each individual by AccuBrain^®^ to indicate the whole brain AD-pathological atrophy degree. The AD-RAI ranges from 0 to 100%, representing the severity of brain atrophy. It was calculated according to the atrophy degree of AD-related brain structures, including subcortical structures (e.g. hippocampus), ventricles, and also the cortical lobar regions. Based on an in-house training database with the brain volumetric data of both normal subjects and AD patients, AccuBrain^®^ computes and selects the most relevant brain regional volumetry and projects the multi-dimensional brain regional volumetry features into a single atrophy index (i.e. AD-RAI) for the individual to be tested. Here, the in-house training database contains brain MRI scans of 400 subjects, with 45% AD patients and 55% NC subjects. Regarding the inclusion criteria of the in-house training database, for the AD group they were: (1) diagnosis of AD according to the International Classification of Diseases, 10^th^ Revision (ICD-10), (2) CDR≥1, (3) not having any nootropic drugs, such as anticholinesterase inhibitors, and (4) able to perform the neuropsychological test and tolerate the MRI scanning. The inclusion criteria for the NC group were: (1) normal in general physical status, (2) a CDR of 0 and (3) no memory complaints.

### Statistical analyses

We compared the demographic characteristics of the five groups of subjects (NCs, MCIs, ADs, NCc and MCIc) using ANOVA with Bonferroni correction for between-group comparisons. The AD-RAIs of the five groups were also compared with ANOVA to confirm their consistence with the diagnosis of the subjects. In addition, Pearson partial correlation analyses were performed to associate baseline AD-RAI with CSF-based/PET-based biomarkers (with age and gender as covariates) and cognitive measures (with age, gender and education level as covariates) over the two years. Subsequently, we focused on the brain volumetric difference between converters and non-converters (e.g. NCc vs. NCs) identified by the baseline measures and the longitudinal changes over the two years.

### Baseline brain volumetry in converters and non-converters

ANCOVA analyses were first performed to identify the measures of baseline brain volumetry (measures of single regions and the AD-RAI based on multiple regions) that presented significant difference between NCs and NCc, and between MCIs and MCIc respectively. Age and gender were covaried out in these comparisons. Using the baseline brain volumetry measures that were significantly different between converters and non-converters, we further performed logistic regression analyses, where the condition of conversion (e.g. NCc vs. NCs) was the dependent variable, with a dichotomized brain volumetry measure as the independent variable. To compare the performance of these baseline measures of brain volumetry in differentiating converters and non-converters, we also optimized the cutoff when dichotomizing a volumetric measure (the independent variable) to achieve the best area under the curve (AUC) of receiver operating characteristics (ROC) for the corresponding measure. Here, the candidate cutoff values were selected for different type of volumetric measures.

Regarding the AD-RAI (ranging from 0 to 1), we searched within the range of 0.1~0.9 in increments of 0.1 for the best cutoff, where the cutoffs were determined by the exact value of this index. Different from AD-RAI which indicates severity of AD-like atrophy pattern, the exact values of the volumetric measures of individual regions do not represent atrophy degree, and the ranges of their exact values vary for different brain regions. To dichotomize these volumetric measures with similar criteria, we applied percentiles (based on the data of this study) as the cutoffs. As the expected “norms” of volumetric measures may vary for NC-to-MCI conversion and MCI-to-AD conversion, we calculated the percentiles of the volumetric measures of individual regions separately for NC group (including subjects of NCs and NCc) and MCI group (including subjects of MCIs and MCIc). For lobar atrophy measures and ventricle volumes which are expected to positively associate with the risk of disease progression, we first selected median (50^th^ percentile) and 75^th^ percentile as the cutoffs, which have been widely used in literature [39]. Furthermore, we chose 90^th^ percentile instead of even higher ones (e.g. 95^th^ percentile) as the cutoff of upper limit, aiming to leave at least 10% of the data to the smaller group (dichotomized by volumetric measures) due to the small sample size of our study (n=73 for NC group and n=85 for MCI group). Finally, we considered 80^th^ and 85^th^ percentiles for a finer searching within the upper range (75%~90%). In this regard, the candidate cutoffs for lobar atrophy measures and volumetric measures of ventricle structures were 50^th^, 75^th^, 80^th^, 85^th^ and 90^th^ percentiles. Similarly, for the volumetric measures of subcortical structures which are expected to negatively associate with the risk of disease progression, the candidate cutoffs were 10^th^, 15^th^, 20^th^, 25^th^ and 50^th^ percentiles. These cutoffs were subsequently used in the logistic regressions for the analyses of NCs vs. NCc and MCIs vs. MCIc.

### Longitudinal trajectory of brain volumetric changes of converters and non-converters

As the data at 6 and 12 months before the last diagnostic visit (at 24 months) was also available for most of the subjects, it should also be interesting to map the difference of longitudinal trajectory of brain volumetric changes between the converters and non-converters (e.g. NCs vs. NCc). Here, we used linear mixed effect model, which can properly account for correlation between repeated measurements on the same subject and handle missing data more appropriately than the traditional repeated ANOVA analyses [40]. The interaction effect of group × time was tested with age and gender as covariates, where the group variable (independent variable) was defined as conversion status during the two years (for baseline NC subjects or MCI subjects), and the dependent variables were the volumetric measures (measures of single regions and the AD-RAI based on multiple regions) at different timepoints. With the mapping of longitudinal trajectory, we could also identify the brain regional volumetric measures that provided clues of further disease progression on top of the baseline measures, for example, if NCc subjects had more rapid atrophy at 6 or 12 months than NCs subjects.

## Supplementary Material

Supplementary Tables
