# Supplementary material for: Risk estimation before progression to mild cognitive impairment and Alzheimer’s disease: an AD resemblance atrophy index
Source: Aging (Albany NY). 2019 Aug 29;11(16):6217–36. doi: 10.18632/aging.102184 (PMC6738429; doi:10.18632/aging.102184)
Supplement: Supplementary Tables [file aging-11-102184-s001.pdf]

SUPPLEMENTARY TABLES

Supplementary Table 1. Available number of subjects for biomarkers of CSF and F18-AV-45 PET.

	NCs	NCc	MCIs	MCIc	AD	Total
Included subjects^, n	50	23	50	35	25	183
CSF Aβ_{42}, n						
Baseline	44	12	48	35	25	164
24 months	27	6	31	20	12	96
CSF t-tau, n						
Baseline	44	12	48	34	23	161
24 months	27	6	31	19	12	95
CSF p-tau$_{181}$, n						
Baseline	44	12	48	35	25	164
24 months	27	6	31	20	12	96
Mean cortical SUVR*, n						
Baseline	50	23	50	35	25	183
24 months	48	18	42	34	22	164

^Total number of the subjects included in this study. *Mean average cortical uptake (within frontal, anterior/posterior cingulate, lateral parietal, and lateral temporal cortex) of Florbetapir (F18-AV-45) PET with the whole cerebellum as the reference region. NCs, NC stable subjects; NCc, NC-to-MCI converters; MCIs, MCI stable subjects; MCIc, MCI-to-AD converters; ADs, AD stable subjects; CSF, cerebrospinal fluid; SUVR, standard uptake value ratio.

Supplementary Table 2. MoCA and its subscores at baseline and 24 months.

	NCs (n=50)	NCc (n=23)	MCIs (n=50)	MCIc (n=35)	ADs (n=25)	*p*
MoCA, mean (SD)						
Baseline	25.98 (1.76)	25.13 (2.10)	24.62 (2.39)	23.49 (2.72)	19.52 (5.10)	<0.001
24 months	26.46 (1.61)	25.55 (1.87)	25.02 (2.56)	19.86 (4.10)	14.21 (6.20)	<0.001
MoCA-memory, mean (SD)						
Baseline	9.58 (1.03)	9.52 (1.08)	9.44 (1.25)	9.20 (1.35)	7.32 (2.76)	<0.001
24 months	9.64 (0.72)	9.55 (0.74)	9.66 (1.30)	6.97 (2.05)	5.00 (2.75)	<0.001
MoCA-visuospatial, mean (SD)						
Baseline	3.42 (0.73)	3.00 (0.90)	3.26 (0.83)	2.94 (0.97)	2.44 (1.12)	<0.001
24 months	3.62 (0.60)	3.14 (0.94)	3.10 (0.86)	2.57 (1.07)	1.79 (0.98)	<0.001
MoCA-language, mean (SD)						
Baseline	4.58 (0.61)	4.61 (0.66)	4.30 (0.76)	4.17 (1.07)	3.92 (1.08)	0.007
24 months	4.76 (0.52)	4.59 (0.59)	4.38 (0.81)	3.60 (1.24)	2.96 (1.46)	<0.001
MoCA-attention, mean (SD)						
Baseline	3.86 (0.45)	3.70 (0.56)	3.64 (0.56)	3.34 (0.80)	2.64 (1.35)	<0.001
24 months	3.92 (0.34)	3.82 (0.39)	3.62 (0.67)	3.03 (0.98)	1.96 (1.52)	<0.001
MoCA-executive, mean (SD)						
Baseline	4.54 (0.65)	4.30 (0.88)	3.98 (1.00)	3.83 (1.15)	3.20 (1.32)	<0.001
24 months	4.52 (0.95)	4.45 (0.80)	4.26 (0.90)	3.69 (1.21)	2.50 (1.44)	<0.001

NCs, NC stable subjects; NCc, NC-to-MCI converters; MCIs, MCI stable subjects; MCIc, MCI-to-AD converters; ADs, AD stable subjects.
